# A Rare Case of the Simultaneous, Multifocal, Metastatic Renal Cell Carcinoma to the Ipsilateral Left Testes, Bladder, and Stomach

**DOI:** 10.1155/2016/1829025

**Published:** 2016-01-21

**Authors:** Michael Kongnyuy, Samuel Lawindy, Daniel Martinez, Justin Parker, Mary Hall

**Affiliations:** ^1^University of South Florida Morsani College of Medicine, Tampa, FL 33612, USA; ^2^James A. Haley Veterans' Hospital, Tampa, FL 33612, USA

## Abstract

We describe the rare case of a 68-year-old gentleman with the history of a hand-assisted laparoscopic left radical nephrectomy for a T2bN0M1 clear cell renal cell carcinoma (RCC). Seven years after surgery and with clean surveillance imaging for metastasis/recurrence the patient presented with three separate tumors suspicious for malignancy. A bladder lesion was found during workup for hematuria, a stomach lesion during diagnostic endoscopy, and a testicular lesion during self-exam. He underwent transurethral resection of bladder tumor, left inguinal orchiectomy, and upper endoscopic ensnarement. All specimens surprisingly showed RCC by histology and immunostaining. These three sites are rare for RCC metastasis and simultaneous presentation is even rarer, further emphasizing the importance of continuous and careful follow-up in this patient population, despite what could appear as complete remission.

## 1. Introduction

Renal cell carcinoma has a strong potential to metastasize to multiple organs [1]. The most common sites of renal cell carcinoma metastasis are the liver, brain, bones, and lung. Stomach metastasis is very rare (0.2–4%) and testicular metastasis even rarer (0.1–1%) [2, 3]. The time to metastasis generally occurs years beyond primary cancer presentation. In testicular metastasis, it usually occurs simultaneously or precedes the diagnosis of renal tumors [4]. We report a rare case of simultaneous presentation of RCC metastasis to the stomach, bladder, and ipsilateral left testicle 7 years after left nephrectomy for clear cell renal cell carcinoma (RCC).

## 2. Case Report

**Figure 1:**
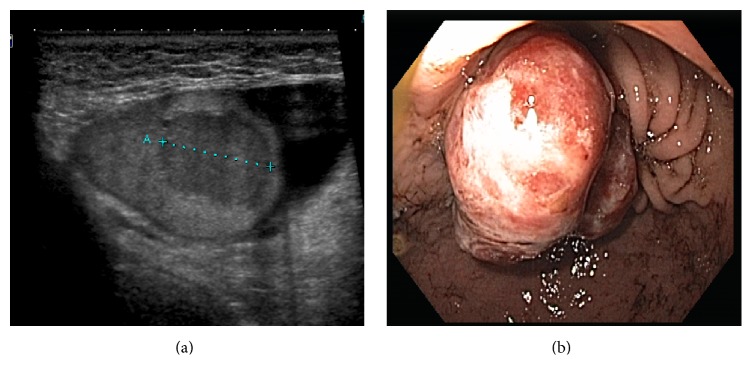
(a) Ultrasound showing testicular metastasis of RCC; mass measuring 2.1 × 1.7 × 2.0 cm. (b) Endoscopy showing metastatic RCC mass to the fundus of the stomach.

A 68-year-old gentleman with a history of T2bN0M1 clear cell RCC status after hand-assisted laparoscopic left radical nephrectomy (no lymph node dissection), 7 years ago, without evidence of recurrence on surveillance imaging presented with a palpable left testicular mass. During follow-up of his RCC with serial scans, he was found with hematuria and transurethral resection of the bladder specimen was positive for papillary urothelial neoplasm of low malignant potential (PUNLMP). Patient was then followed with serial scans and cystoscopies (and cell cytology) for RCC and PUNLMP, respectively. During follow-up, patient presented to his primary care physician (PCP) with a painless, left testicular mass noted on self-examination. Given his history of RCC, his PCP asked that he be further evaluated by a urologist. Testicular ultrasound showed a 2.1 × 1.7 × 2.0 cm heterogeneous, hypoechoic lesion in the left testicle, which was highly suspicious for a testicular neoplasm (Figure 1(a)). He was scheduled for a left inguinal orchiectomy.

During his preoperative workup, he was noted to be anemic and was found to have black tarry stools. A gastroenterologist was consulted and an upper endoscopy showed a friable, ulcerated, and nodular mass in the fundus of the stomach (Figure 1(b)). Two months before, during routine endoscopy, a polyp was noted, biopsied, but the results were inconclusive; however during the second endoscopy the pathology results showed clear cell RCC.

A cystoscopy, prior to starting the left inguinal orchiectomy, was done (given his history of PUNLMP) and showed a small subcentimeter hyperemic nodular area in the bladder base, just anterior to the trigone, which was biopsied. The radical orchiectomy was performed without any complications after biopsy of the bladder nodule. Pathology reported clear cell RCC with positive CD10, CK8/18 immunochemistry for both testicular and bladder specimens.

## 3. Discussion

It is a well-established fact that RCC, like melanoma, is a clinical chameleon as it can metastasize to just about any organ in the body [1–10]. With common sites of metastasis being the lungs, bones, liver, and brain, RCC still remains quite unpredictable in its pattern of spread [1, 2, 4, 5, 7, 10]. When secondary testicular cancer of renal origin occurs, it usually presents simultaneously or within a year from primary RCC presentation [5]. While gastrointestinal (GI) metastasis of RCC is slightly more common than testicular metastasis, it remains an unusual site of RCC metastasis [2, 8]. Testicular metastasis of RCC ranges from 0.1 to 1% while GI metastasis is at 0.1–4% [1–8]. Often, GI metastasis presents with GI bleeding, anemia, and dysphagia [3, 6–8].

Testicular metastasis of RCC often occurs in the ipsilateral left testicle and generally precedes or concurrently presents with primary RCC. Rarely have there been cases of delayed testicular metastasis of RCC [5, 9]. Our patient presents with a unique case of delayed ipsilateral left testicular metastasis of RCC simultaneously occurring with GI and bladder metastasis of RCC. Like any other GI metastatic tumor, patient complained of black tarry stools, fatigue, and dysphagia. Incidentally, on cystoscopy, a bladder lesion was noted and resected. The testicular mass and bladder lesion were asymptomatic as patient denied current frank hematuria, frequency, or testicular tenderness.

RCC (T2bN0M1) metastasis in our patient was a surprise due to the distant nature of the original surgery and it remains significant to note that all three lesions occurred simultaneously and could impact his prognosis. However, this combination presentation of simultaneous multifocal metastasis of RCC has not been reported to the best of our literature search, which was done, on PubMed using search words such as “renal cell carcinoma metastasis,” “gastric metastasis,” “testicular metastasis,” “simultaneous,” and “delayed.” This was an unfortunate patient and it goes to solidify the capricious nature of RCC but most importantly add to the library of case reports of rare testicular metastasis of RCC.

## 4. Conclusion

RCC metastasis to the testicle, genitourinary, and gastrointestinal tract remains a rarity and simultaneous multifocal RCC metastasis to these sites is even rarer. It goes without saying that urologists and oncologists should anticipate just about any pattern of presentation of RCC metastasis, and this report emphasizes the importance of close follow- up and a high suspicion for odd areas of metastasis.
